# Spreading Dynamics from Social-Network Models to Complex Agricultural Systems: A Mini-Review

**DOI:** 10.3390/e28101080

**Published:** 2026-09-30

**Authors:** Jiao Wu, Xiuwen Feng, Lihui Zhu, Yingpeng Liu, Muhua Zheng

**Affiliations:** 1 School of Mathematical Sciences, Jiangsu University, Zhenjiang 212013, China; wujiao@ujs.edu.cn (J.W.);; 2 School of Physics and Electronic Engineering, Jiangsu University, Zhenjiang 212013, China

**Keywords:** spreading dynamics, complex networks, complex agricultural systems, theoretical approaches

## Abstract

Spreading dynamics have been extensively studied in social, communication, and epidemiological networks, leading to substantial advances in understanding spreading thresholds, outbreak patterns, temporal dynamics, and phase transitions. However, their extension to other complex systems, particularly agricultural systems, remains less systematically explored. In this mini-review, we summarize representative spreading models and theoretical approaches developed for complex networks. We first review spreading dynamics on single-layer networks and then discuss their extensions to multilayer, temporal, and higher-order networks, highlighting how increasingly complex interaction structures affect spreading thresholds, outbreak patterns, and phase transitions. We subsequently examine how these models and theoretical approaches can be transferred from social networks to complex agricultural systems. Representative applications include crop diseases and pests, livestock diseases, agricultural information and technology diffusion, and coupled disease–information processes. Finally, we discuss emerging directions for extending spreading theories to increasingly complex agricultural systems. This review may help clarify the generality and transferability of spreading theories across different networked systems and stimulate their further development in complex agricultural contexts.

## 1. Introduction

Spreading phenomena are ubiquitous in complex systems [1,2,3], including epidemic spreading, information diffusion, behavioral adoption, and cascading failures. Although these processes differ in their physical mechanisms, they exhibit common dynamical features in which local interactions among individuals or components collectively shape large-scale spreading behavior [4,5]. Understanding these spreading mechanisms is essential for predicting spreading outcomes and developing effective intervention strategies, such as controlling epidemic outbreaks, mitigating misinformation propagation, promoting beneficial information diffusion, and enhancing the resilience of networked systems.

In many real-world systems, spreading processes take place over networks of interactions among individuals, groups, or functional units [6,7]. These interactions determine the available pathways for transmission and therefore play a crucial role in shaping spreading dynamics. Consequently, considerable effort has been devoted to understanding how network structure affects spreading thresholds, outbreak sizes, propagation patterns, and dynamical transitions [1,8]. Over the past decades, numerous spreading models and theoretical approaches have been developed to characterize these processes [7,8]. Classical compartmental, threshold, rumor, and social contagion models provide fundamental descriptions of different spreading mechanisms, while analytical approaches such as mean-field theory, heterogeneous mean-field theory, message-passing methods, and edge-based compartmental theory have been widely used to analyze their dynamical behaviors [8]. Early studies mainly focused on single-layer networks, whereas subsequent research has increasingly considered more complex interaction structures, including multilayer [9,10,11], temporal [12], and higher-order networks [13,14,15].

Despite substantial progress in spreading dynamics, existing reviews have mainly focused on social, communication, and epidemiological networks [1,2,3]. Meanwhile, the development of theoretical approaches for increasingly complex network structures has substantially broadened the scope of spreading research [1,8]. The extension of these models and analytical approaches to other complex systems, however, remains less systematically explored. Complex agricultural systems provide a particularly relevant example, as biological transmission, transportation, information diffusion, behavioral adoption, and control processes may coexist and interact across interconnected networks [16,17,18,19].

Agricultural spreading processes are highly heterogeneous and involve distinct interaction mechanisms. Crop diseases and pests may spread through spatial proximity, environmental dispersal, and transportation among fields or regions [20,21,22,23], whereas livestock diseases are strongly influenced by animal contacts and time-varying farm-to-farm movements [24,25,26]. By contrast, agricultural information and technology adoption depend more strongly on farmer social networks, peer learning, and behavioral reinforcement [27,28,29]. These differences motivate the use of different spreading frameworks, ranging from susceptible-infected-susceptible (SIS) and susceptible-infected-recovered (SIR) models and spatial-network models for biological transmission to temporal-network models for livestock movements and threshold or complex-contagion models for behavioral adoption.

Existing studies, including empirical analyses and data-informed modeling, already demonstrate the potential of these approaches in diverse agricultural systems. Spatially embedded and metapopulation models have been used to characterize crop-disease transmission and landscape-mediated spreading [20,21,30]. Spatial and agent-based approaches have also been applied to pest dispersal and invasion across heterogeneous landscapes and transportation networks [22,23,31]. In livestock systems, empirical animal-movement networks have been used to study farm-to-farm disease transmission and targeted surveillance or control [24,25,26]. Farmer social networks have further been shown to influence both agricultural technology adoption and information diffusion [27,28,29]. More recently, multilayer approaches have begun to connect biological transmission with information sharing and behavioral responses, highlighting the potential importance of coupled disease–information dynamics in agricultural risk management [32,33].

These applications also reveal important practical limitations. Agricultural interaction data are often incomplete, heterogeneous, and collected at different spatial or temporal resolutions, which complicates network reconstruction and model calibration. In particular, high-resolution temporal contacts and group interactions required for temporal and higher-order network models remain relatively scarce. This creates a gap between increasingly sophisticated spreading theories and their empirical implementation in agricultural systems.

Against this background, this mini-review examines how spreading models and theoretical approaches developed for social and contact networks can be extended to complex agricultural systems. We review spreading dynamics from single-layer to multilayer, temporal, and higher-order networks, and then discuss their applicability, empirical implementations, and practical limitations across representative agricultural scenarios [34,35,36,37,38]. Finally, we highlight current challenges and future research directions for spreading dynamics in complex agricultural systems.

## 2. Spreading Models and Theoretical Approaches for Single-Layer Networks

### 2.1. Simple and Complex Contagion

Complex networks provide a general framework for representing complex systems [6]. A network is typically described by a graph G=(V,E), where nodes denote interacting units and edges represent contacts, transportation, communication, or spatial proximity [1,6]. Spreading dynamics on networks can generally be classified into simple and complex contagion according to how transmission depends on exposures from neighboring nodes [39,40]. In simple contagion, a single exposure may be sufficient to trigger transmission, whereas complex contagion typically requires multiple or repeated exposures before adoption or activation occurs [39,40,41,42,43].

Simple contagion is widely used to describe epidemic transmission and information diffusion [44]. Classical examples include the susceptible-infected-susceptible (SIS) and susceptible-infected-recovered (SIR) models, which describe recurrent and non-recurrent spreading processes, respectively [1]. Their dynamics depend strongly on network structure, particularly degree heterogeneity and connectivity patterns, which can substantially affect spreading thresholds, outbreak sizes, and propagation speed [1,7].

By contrast, complex contagion describes spreading processes in which transmission or adoption depends on multiple or repeated exposures [39,40,41,42,43]. Such mechanisms are particularly relevant to behavioral adoption, social reinforcement, and information diffusion. Typical models include threshold-based and reinforcement-based contagion models, which can exhibit nonlinear spreading behaviors, discontinuous transitions, and bistability [39,40,41,42,43].

This distinction is particularly relevant to agricultural systems. Crop pests and plant diseases may often be described using simple-contagion frameworks, whereas the adoption of agricultural technologies or control practices may require repeated information exposure, peer confirmation, and social reinforcement [20,21,27,28].

### 2.2. Theoretical Approaches to Spreading Dynamics

A variety of theoretical approaches have been developed to analyze spreading dynamics on complex networks, including mean-field (MF), heterogeneous mean-field (HMF), quenched mean-field (QMF), dynamical message-passing (DMP), pairwise approximation (PA), bond percolation (BP), and edge-based compartmental theory (EBCT) [1,8]. Table 1 summarizes the main assumptions, structural resolution, agricultural applicability, and limitations of these representative approaches.

These approaches differ mainly in the amount of structural and dynamical information retained. MF provides the simplest macroscopic approximation by treating nodes as statistically equivalent and neglecting network heterogeneity [45]. HMF incorporates degree heterogeneity by grouping nodes according to their degrees [44], while QMF retains node-level structural information through the adjacency matrix [46]. DMP describes transmission through directed-edge messages and suppresses immediate backtracking, thereby partially accounting for dynamical correlations [47,48]. PA further tracks the joint states of connected node pairs, providing a more detailed description of local dynamical correlations at the cost of increased computational complexity [49,50]. For irreversible spreading processes such as the SIR model, BP provides a useful mapping between epidemic spreading and network connectivity and is particularly suited to predicting the final outbreak size [51]. These methods involve different trade-offs between analytical tractability, structural resolution, and the treatment of dynamical correlations [8].

**Figure 1:**
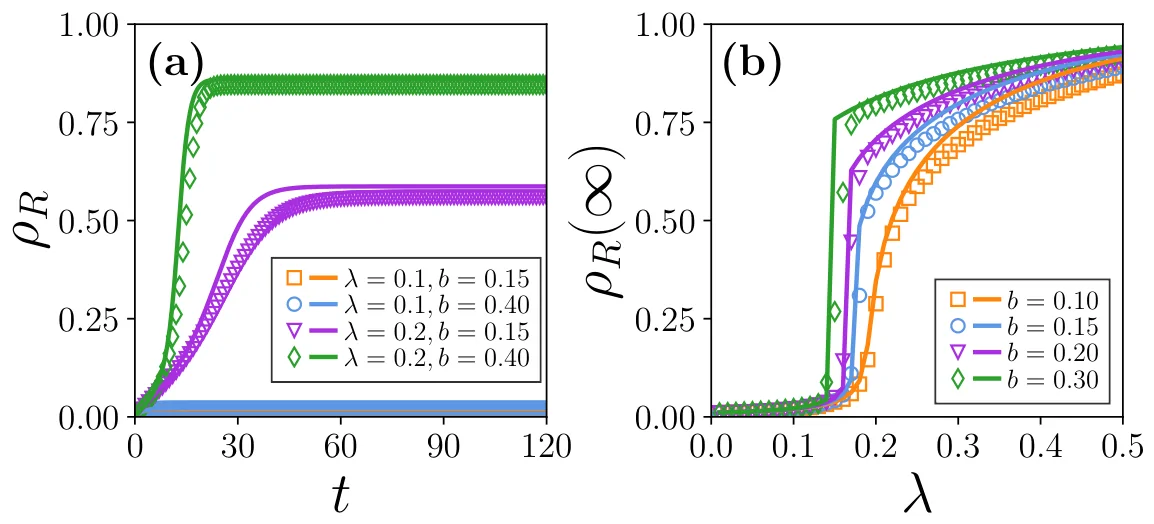
(Color online). Edge-based compartmental theory for complex contagion on a single-layer network. (**a**) Time evolution of the recovered fraction $\rho_{R}\left(t\right)$ for different information sensitivities λ and social reinforcement strengths *b*. (**b**) Final spreading size $\rho_{R}\left(\infty \right)$ as a function of the information sensitivity λ. Symbols represent numerical simulations, while solid lines denote the predictions of the edge-based compartmental theory. Reproduced from Ref. [62] with permission.

Among these approaches, edge-based compartmental theory (EBCT) provides a flexible framework for describing spreading dynamics from the perspective of transmission along edges and is particularly suitable for irreversible processes such as the SIR model [52,53,54,55,56,57,58,59]. By introducing edge-based transmission probabilities and a cavity-state formulation, EBCT can capture both the temporal evolution and the final spreading size while partially accounting for dynamical correlations. Importantly, EBCT is not limited to simple contagion. It can also be extended to complex contagion, in which adoption depends on repeated exposures, cumulative information, or social reinforcement [53,60,61]. Such extensions can describe non-Markovian spreading processes and nonlinear dynamical behaviors, including discontinuous transitions and bistability [53,62]. As illustrated in Figure 1, EBCT successfully reproduces both the temporal evolution and the final spreading size of a reinforcement-based social contagion process [62].

As summarized in Table 1, from an agricultural perspective, the applicability of these approaches depends strongly on the scale and structural characteristics of the system. Mean-field and compartmental approaches are useful for describing aggregate population-level spreading, but their homogeneous-mixing assumption may be inappropriate for geographically dispersed and structurally heterogeneous farms [1,16,17,45]. Degree-based approaches can capture part of this heterogeneity [8,44], whereas node- and edge-level methods are more suitable when empirical contact, movement, or trade networks are available [24,26,46,47]. However, many of these approaches assume a static interaction structure, which may become inadequate when agricultural contacts, transportation, or animal movements vary seasonally or over time [12,63,64]. These limitations motivate the use of more complex representations, such as temporal and multilayer networks, discussed in the following sections [9,11].

## 3. Spreading Dynamics on Multilayer Networks

### 3.1. Multilayer Network Structure

Many real-world systems involve multiple types of interactions that cannot be fully represented by a single network [9,10,11]. Multilayer networks provide a general framework for describing such systems by organizing interactions into multiple interconnected layers, where each layer may represent a distinct type of interaction, communication channel, transportation pathway, or functional subsystem. Nodes can interact within the same layer through intra-links and across different layers through inter-links, thereby capturing multiple communication channels within a unified network framework [9,10]. Common forms of multilayer networks include multiplex networks, where the same nodes appear across different layers, and networks of networks, where distinct networks are interconnected through inter-network links [11].

### 3.2. Coupled Spreading Processes

Spreading processes on multilayer networks can occur through multiple interaction channels and are influenced by both intra- and interlayer connections [9,10,11,65,66,67,68]. A common setting considers the same spreading process propagating across different layers, where each layer represents a distinct communication or contact channel. Transmission within individual layers follows conventional spreading rules, while interlayer links allow the process to propagate between layers. Compared with single-layer networks, coupling between layers can substantially alter the spreading threshold, outbreak size, and propagation pattern [67,68].

**Figure 2:**
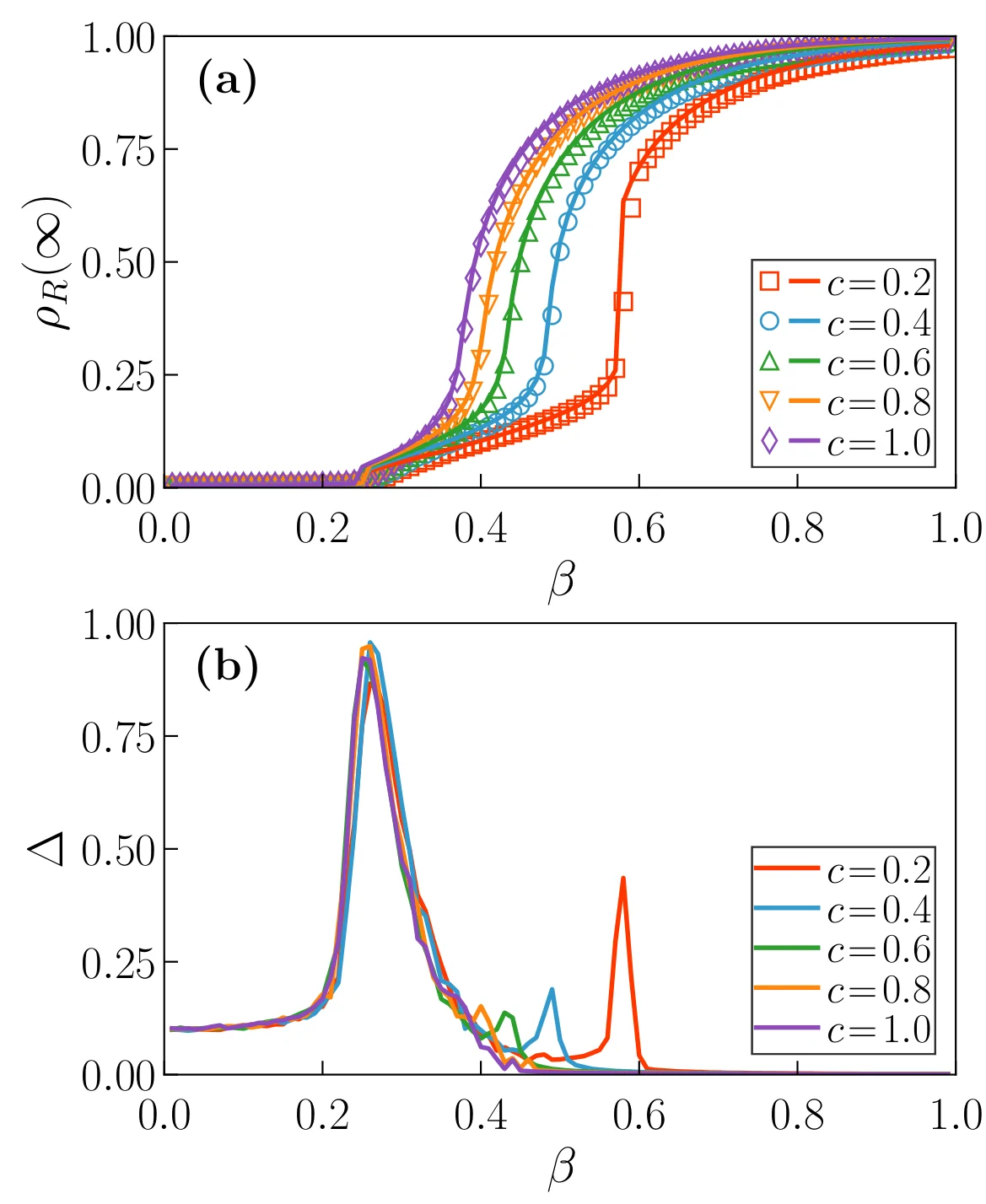
(Color online). Interlayer asymmetry induces double transitions in a two-layer network. (**a**) Final recovered fraction $\rho_{R}\left(\infty \right)$ as a function of the transmission probability β for different interlayer asymmetries *c*. Symbols represent numerical simulations, while solid lines denote the predictions of the edge-based compartmental theory. (**b**) Variability Δ as a function of β, identifying the corresponding transition points. Adapted from Ref. [69] with permission.

For example, our previous studies revealed several characteristic spreading phenomena induced by interlayer coupling. Weak coupling can separate the spreading timescales of different layers, leading to localized spreading or multiple epidemic peaks, whereas stronger coupling tends to synchronize outbreaks across layers [66,68]. For complex contagion, the interplay between interlayer coupling and reinforcement effects can generate double transitions [67]. Interlayer asymmetry further modifies the activation order and transition behavior of different layers and can induce pronounced double spreading transitions, as illustrated in Figure 2 [69].

**Figure 3:**
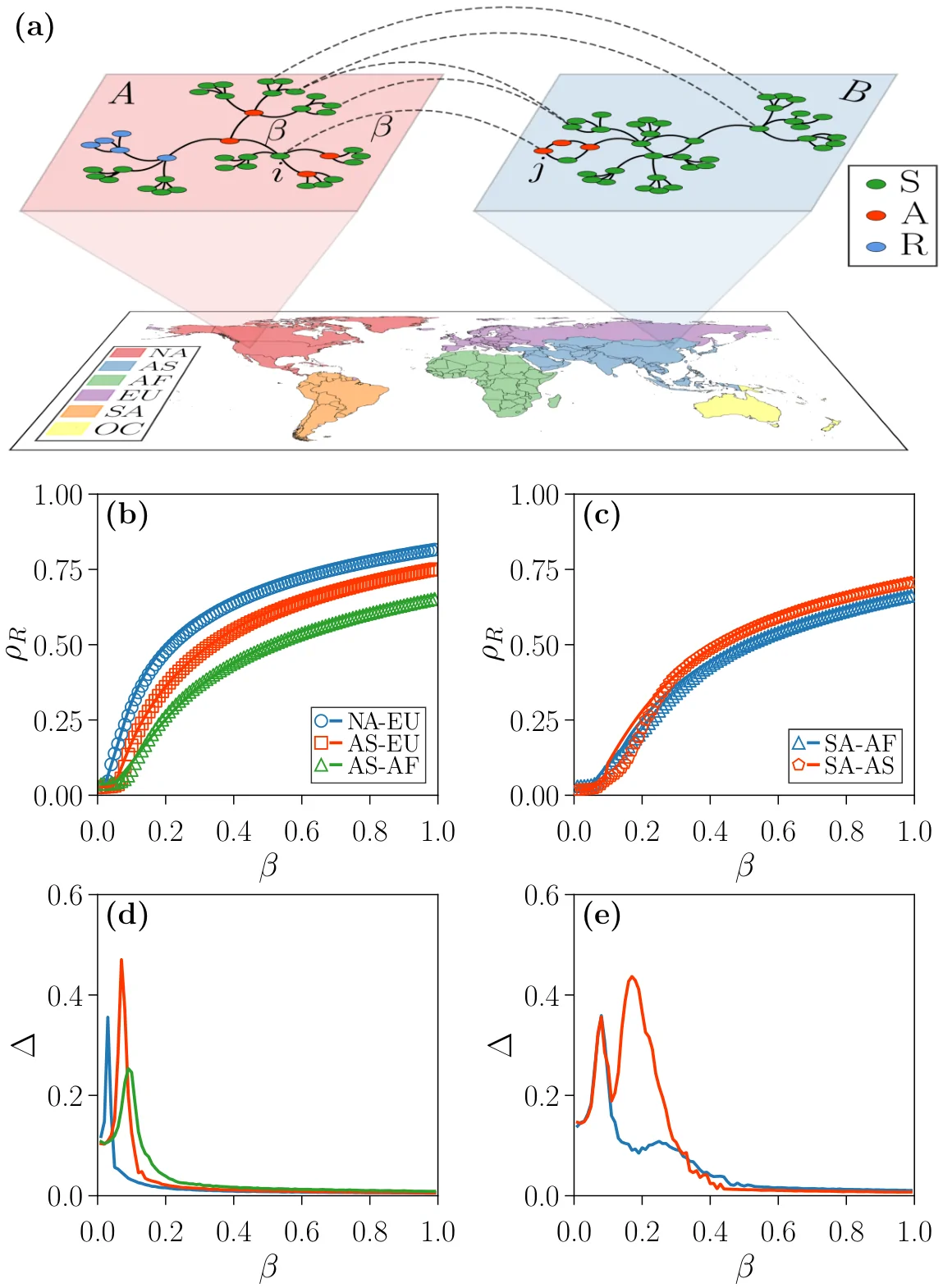
(Color online). Self-similar continental networks promote synchronous information outbreaks, whereas weak inter-continental connectivity induces double transitions. (**a**) Schematic illustration of information spreading between two interconnected continental networks. Networks *A* and *B* represent communication networks from two continents, with solid and dashed edges denoting intra- and inter-continental links, respectively. Information is transmitted along each edge with probability β. (**b**,**c**) Final recovered fraction $\rho_{R}$ as a function of the transmission probability β for different pairs of continental networks. Symbols represent numerical simulations, while solid lines denote the predictions of the edge-based compartmental theory. (**d**,**e**) Corresponding variability Δ, showing a single transition for well-connected continental networks and double transitions for weakly connected networks. The line colors correspond to those used in panels (**b**,**c**). Adapted from Ref. [70] with permission.

More recently, we showed that structural similarity between layers promotes synchronous outbreaks, whereas sufficiently weak interlayer connectivity favors the emergence of double transitions [70]. This behavior is observed in continental-scale Twitter communication networks, where most well-connected continental pairs exhibit a single transition, while sparsely connected pairs display double transitions, as shown in Figure 3. These results further demonstrate that spreading patterns in multilayer networks depend not only on coupling strength but also on the structural organization of the interacting layers.

Multilayer networks can also support interacting spreading processes, in which different dynamical processes evolve on different layers and influence each other. A representative example is the coupled spreading of epidemics and awareness. Granell et al. showed that awareness diffusion can suppress epidemic spreading and identified a metacritical point separating regimes in which awareness does or does not affect the epidemic threshold [71]. Related studies further demonstrated that information spreading can reduce disease prevalence, while epidemic outbreaks can in turn stimulate information diffusion [72]. Interacting contagions may also cooperate with each other; Cai et al. found that sufficiently strong cooperation can produce abrupt outbreaks and hybrid phase transitions [73]. These studies show that interactions between spreading processes can substantially modify epidemic thresholds, outbreak sizes, and transition behavior.

### 3.3. Theoretical Approaches for Multilayer Spreading

Theoretical approaches developed for single-layer networks have been extended to multilayer systems by introducing layer-dependent dynamical variables and coupling between layers [10,74]. Representative methods include mean-field and heterogeneous mean-field theories, spectral approaches based on supra-contact matrices, message-passing methods, percolation theory, and edge-based compartmental theory [9,10,11]. Here, we focus on the multilayer extension of EBCT, which explicitly distinguishes transmission through different intra- and interlayer connections [62,66,67,68,69,70].

In a two-layer network, let $\theta_{x,y}\left(t\right)$ denote the probability that a node in layer *x* has not received information through an edge from a neighboring node in layer *y* by time *t*, where x,y∈{1,2}. The four variables $\theta_{1,1}$, $\theta_{1,2}$, $\theta_{2,1}$, and $\theta_{2,2}$ therefore describe transmission through the different intra- and interlayer channels. This formulation extends single-layer EBCT by allowing transmission and accumulated exposures to depend on the layer through which they occur [66,67].

For complex contagion, a susceptible node may accumulate exposures through multiple channels. If a node has *k* neighbors associated with a given channel, the probability of receiving *m* successful transmissions can be written as

(1) τ(k,m,θ)=kmθk−m(1−θ)m.

The total probability that a node remains susceptible can then be obtained by combining exposures received through the different intra- and interlayer connections, together with the corresponding joint degree distribution and adoption rule.

The evolution of each edge variable is governed by the probability that the neighboring node is active but has not yet transmitted through the focal edge. In a general form,

(2) $$\frac{d\theta_{x,y}\left(t\right)}{dt}=-\beta_{x,y}\Phi_{x,y}^{A}\left(t\right),$$

where $\beta_{x,y}$ is the transmission rate from layer *y* to layer *x*, and $\Phi_{x,y}^{A}\left(t\right)$ denotes the probability that the neighboring node is active but has not yet transmitted information through the focal edge.

Once the edge variables are determined, the susceptible fraction in layer *x* can be calculated as

(3) $$S_{x}\left(t\right)=\sum_{k_{1},k_{2}}P_{x}\left(k_{1},k_{2}\right)Y_{x}\left(k_{1},k_{2},t\right),$$

where $P_{x}\left(k_{1},k_{2}\right)$ is the joint degree distribution and $Y_{x}\left(k_{1},k_{2},t\right)$ denotes the probability that a node with joint degree $\left(k_{1},k_{2}\right)$ remains susceptible.

This framework extends single-layer EBCT by explicitly distinguishing intra- and interlayer transmission and accounting for exposures accumulated through multiple channels. It therefore provides a useful approach for describing complex contagion and phase transitions in coupled networks. As shown in Figure 3, the resulting EBCT closely agrees with numerical simulations for both single and double transitions in interconnected continental communication networks [70]. In particular, it captures synchronous outbreaks in well-connected networks and the emergence of two distinct transition points when interlayer connectivity becomes sufficiently weak.

From an agricultural perspective, multilayer approaches are particularly useful when different spreading and interaction processes coexist, such as biological transmission, animal or commodity movement, information exchange, and management responses [32,33]. By separating these processes into distinct but coupled layers, multilayer models can represent interactions that cannot be captured by a single network [9,10,11]. Their applicability, however, depends on several assumptions. The relevant layers and interlayer connections must be identified, and coupling strengths between layers must be estimated from empirical data. In agricultural systems, these quantities are often incompletely observed, heterogeneous across regions or farms, and may vary over time. Consequently, static multilayer representations may become inadequate when transportation, information exchange, or control responses change seasonally or dynamically. These limitations motivate temporal and higher-order extensions, which are discussed in the following section.

## 4. Spreading Dynamics Beyond Multilayer Networks

Beyond multilayer networks, spreading dynamics have also been extensively studied on temporal [12,75] and higher-order networks [13,76,77], which capture two important features that are absent from conventional static pairwise representations. Temporal networks describe interactions that appear, disappear, or change over time, whereas higher-order networks account for interactions involving more than two nodes simultaneously. These extensions provide a more realistic description of many social, biological, and technological systems and can lead to spreading behaviors that differ substantially from those observed on static pairwise networks [12,13].

In temporal networks, the timing and ordering of contacts play a central role in determining whether and how rapidly spreading can occur [78]. Previous studies have shown that temporal structure can substantially modify epidemic thresholds and outbreak sizes compared with static, time-aggregated networks [78,79]. Valdano et al. demonstrated that epidemic thresholds depend on the full sequence of contact patterns through an infection-propagator framework [80]. Activity-driven models further showed that heterogeneous activation, memory, and adaptive contact changes can either facilitate or suppress spreading by shifting the epidemic threshold [81,82]. Temporal heterogeneity and burstiness may also alter diffusion speed in nontrivial ways, while modular temporal structure can hinder short-lived outbreaks but favor epidemic persistence under different dynamical regimes [83,84]. These results highlight that temporal ordering, activity heterogeneity, and memory are fundamental determinants of spreading dynamics that cannot generally be captured by static network representations.

Higher-order networks provide another important extension by representing interactions among groups of nodes through hyperedges or simplices [13,14]. Such group interactions are especially relevant to complex contagion, where adoption may depend on simultaneous or repeated exposures from multiple neighbors [41,85]. Iacopini et al. introduced a simplicial contagion model and showed that sufficiently strong higher-order interactions can change a continuous spreading transition into a discontinuous one [76]. Matamalas et al. further demonstrated that higher-order infection through simplices can induce abrupt epidemic transitions in SIS dynamics [77]. More general hypergraph contagion models developed by de Arruda et al. revealed a rich phase diagram characterized by continuous and discontinuous transitions, bistability, and hysteresis [86,87]. These studies demonstrate that group interactions can fundamentally change both the onset and the nature of spreading transitions [88].

Beyond the strength of higher-order transmission, the organization of higher-order structures also plays an important role [89,90]. Our previous work showed that correlations between edge and triangle degrees can promote explosive information spreading [91]. More recently, we found that recurrent groups suppress information spreading in temporal networks, with higher-order interactions further enhancing this effect [92].

Taken together, temporal and higher-order networks extend spreading models by incorporating time-varying and group interactions, providing a more realistic description of real-world spreading processes. Their empirical application in agricultural systems, however, remains relatively limited. Agricultural contacts, movements, and group interactions are often only partially observed, recorded at coarse temporal resolutions, or collected from heterogeneous data sources. These limitations complicate the reconstruction of temporal interaction sequences and higher-order structures, as well as model calibration and empirical validation. In addition, structural failures, such as node or link disruptions, can alter network connectivity and transmission pathways, thereby affecting spreading dynamics. Such effects are also relevant to agricultural networks, where failures or targeted interventions may reshape diffusion pathways [93,94].

## 5. From Social-Network Models to Complex Agricultural Systems

Many spreading processes in agricultural systems can be naturally represented using network frameworks originally developed for social and contact systems [16,17,18,19,63,64,95,96,97,98]. In such a mapping, nodes may represent farms, fields, animals, agricultural facilities, production regions, farmers, or other functional units, while edges describe potential pathways of interaction, such as physical contact, transportation, trade, spatial proximity, or information exchange [99,100,101]. For biological spreading, these links may represent pathways through which pathogens, pests, or invasive species move between plants, fields, farms, or regions [16,17,20,102], while transportation and trade networks provide additional routes through the movement of animals and agricultural products [16,17]. For information and behavioral spreading, social relationships among farmers and agricultural organizations determine how knowledge, warnings, technologies, and management practices diffuse through agricultural communities [103,104,105].

Agricultural systems exhibit several structural and dynamical features that distinguish them from many conventional social or contact networks. Farms and fields are geographically dispersed and strongly heterogeneous in size, production type, management practice, and environmental conditions, which naturally motivates spatially embedded and weighted network representations [20,21,22]. Agricultural contacts and movements are also highly seasonal and time dependent, requiring temporal networks to preserve the ordering and duration of interactions [24,26,63]. Moreover, biological transmission, animal or commodity movement, information exchange, and management responses often occur through different but interacting channels, motivating multilayer network architectures [32,33]. Group activities, such as shared facilities, markets, or coordinated farming practices, may further generate higher-order interactions that cannot be fully represented by pairwise links [13,14]. These characteristics suggest that realistic agricultural spreading models often require extensions beyond static single-layer networks.

At the microscopic level, agricultural spreading processes are governed by distinct local interaction mechanisms. A network representation therefore requires not only specifying the nodes and edges, but also identifying what is transmitted, how transmission occurs between connected units, and how repeated local interactions give rise to system-level spreading dynamics. Simple-contagion models are generally appropriate when a single successful contact may trigger transmission [106,107,108,109,110], whereas complex-contagion models are more suitable when adoption requires repeated exposure, social reinforcement, or information from multiple sources [111,112,113]. Thus, biological spreading is often represented by direct-contact or movement-mediated transmission, whereas the diffusion of agricultural technologies and management practices may depend more strongly on reinforcement, peer influence, and repeated information exposure. Table 2 summarizes these micro-level mechanisms for representative agricultural scenarios, while Table 3 summarizes the applicability and major practical limitations of the corresponding spreading models.

### 5.1. Crop Disease Spreading

Crop diseases can be represented by contact or spatial networks in which nodes correspond to plants, fields, farms, or regions and edges denote potential transmission pathways [16,17,114,115]. Arias et al. modeled plant epidemics on spatially embedded networks and explicitly considered long-range pathogen dispersal [20], while Estrada et al. investigated epidemic spreading on spatially structured random rectangular networks [116]. These studies illustrate how spatial connectivity can be incorporated directly into epidemic models.

More detailed agricultural applications have incorporated additional sources of spatial and environmental heterogeneity. Radici et al. developed a metapopulation framework that integrates landscape heterogeneity to model airborne brown-rot transmission in peach production [21]. McQuaid et al. examined the spatial dynamics and control of crop pathogens with mixed-mode transmission [30], while Pacilly et al. used landscape-scale simulations to evaluate host resistance for controlling potato late blight [117]. Regional disease forecasting has also combined airborne inoculum, environmental conditions, and satellite observations [118]. At broader scales, global cropland connectivity has been identified as an important factor in the invasion of emerging pathogens and pests [119], while management and stochasticity can themselves generate spatial structures that affect epidemic behavior [120].

These studies support the use of SIR/SIS, spatial-network, and metapopulation approaches for crop diseases, but also highlight important practical limitations. Transmission rates are often uncertain, and environmental conditions, landscape structure, host heterogeneity, and management practices can substantially modify the effective spreading network.

### 5.2. Pest Invasion and Dispersal

Pest invasion differs from local disease transmission because dispersal may involve both landscape-mediated movement and long-distance human transportation. Spatial-network and agent-based models are therefore useful for describing pest movement across heterogeneous agricultural landscapes. For example, using spatial network models, Rother et al. showed that forest cover can limit agricultural pest spread by reshaping connectivity among crop patches [22]. Rebaudo et al. used an agent-based framework to study the human-induced spread of potato moth in Ecuador, illustrating how human activities can generate additional dispersal pathways [31].

At larger spatial scales, commodity and trade networks become important. Venkatramanan et al. modeled commodity flows in relation to the spread of *Tuta absoluta* in Nepal [23], while network models have also been used to study post-entry management of invasive pests [121]. International trade data have further been used to forecast the global spread of invasive pests and pathogens [122], and recent analyses have linked temporal changes in trade networks to alien plant-pest introductions [123].

A major limitation in these applications is that actual dispersal pathways are often only partially observed. Pest movements may simultaneously depend on landscape connectivity, biological dispersal, commodity flows, and human-mediated transport, making network-edge definition and parameter estimation difficult.

### 5.3. Livestock Disease Spreading

Livestock systems provide particularly direct examples of spreading on empirical networks, because animal movements generate measurable links between farms. Network SIR models can describe disease transmission through these links, while temporal-network models are especially relevant when the timing and ordering of animal movements determine whether transmission paths are feasible [63,64,95,96,97,98]. Lebl et al. analyzed the German pig-trade network and demonstrated the importance of network activity for infectious-disease spreading [24]. Pomeroy et al. combined host-movement networks with a multilevel disease-transmission model for foot-and-mouth disease in Cameroon, showing how movement information can be incorporated into disease dynamics [25]. Similar approaches have been used to predict African swine fever transmission through pig and carcass movements in Thailand [124], to analyze disease dynamics across multiple animal species and targeted control strategies [125], and to evaluate how changes in contact-network structure affect foot-and-mouth disease transmission [126]. Empirical contact networks have also been shown to influence cattle-disease modeling [127], while live-pig trade networks have been used to assess disease-prevention strategies [128]. Machado et al. further showed that explicitly quantifying pig-movement dynamics can improve targeted surveillance and control planning [26].

Despite the relatively rich empirical basis, movement records may still be incomplete, temporally aggregated, or heterogeneous across farms and regions. These limitations complicate reconstruction of time-resolved contact networks and partly explain why high-resolution temporal and higher-order representations remain less common in agricultural applications.

### 5.4. Agricultural Technology Adoption

Agricultural technology adoption is fundamentally different from biological transmission: receiving information does not necessarily lead immediately to adoption. Farmers may require repeated exposure, evidence from several peers, or sufficient social reinforcement, making threshold and complex-contagion models particularly relevant. Previous studies consistently demonstrate an important role for farmer social networks in technology adoption [129,130,131,132,133,134,135]. For example, studies have examined social-network effects on water-saving irrigation technology [130], information exposure and agricultural technology adoption in northern Uganda [131], peer learning in digital farmer-to-farmer networks [132], and interactions between social networks and extension services [133].

A particularly informative example is provided by Beaman et al., who examined whether network-theory-based targeting could increase agricultural technology adoption [27]. Their results demonstrate the practical importance of identifying appropriate individuals in farmer networks for initiating diffusion and are consistent with the idea that agricultural adoption often involves social reinforcement rather than simple one-contact transmission.

The main challenge for quantitative modeling is that adoption behavior is highly heterogeneous. Individual thresholds, perceived risks, socioeconomic conditions, and the strength of peer influence are difficult to infer directly from observed network structure.

### 5.5. Agricultural Information Spreading

Agricultural information, including technical knowledge, disease warnings, market information, and management recommendations, can spread through farmer social networks, extension services, and digital communication channels. Social-network diffusion models therefore provide a natural framework for describing these processes.

Previous studies have demonstrated the importance of social-network structure in agricultural information diffusion. Beaman and Dillon examined agricultural information diffusion within social networks in Mali and demonstrated that network structure is closely related to how information reaches farmers [28]. Pratiwi and Suzuki similarly studied farmers’ social networks in rural Indonesia and showed their relevance to knowledge acquisition from agricultural training [29]. More recent work has investigated social media for agricultural information dissemination [136], information flows and proximity within cocoa-farmer networks [137], and seed-selection strategies for information diffusion using agent-based models in rural Zambia [138]. Farmer-to-farmer information flows have also been examined in the Kenyan dairy sector [139]. These studies complement earlier evidence that social relationships can shape both agricultural information diffusion and subsequent technology adoption [103,104,105,133].

Unlike animal-movement networks, however, communication networks are often only partially observed. Informal conversations, multiple communication channels, and unrecorded interactions can result in substantial uncertainty in network reconstruction and spreading parameters.

### 5.6. Coupled Disease–Information Interactions

Disease spreading in agricultural systems is often coupled with the diffusion of information, awareness, and control behaviors. Such interactions can be represented by multilayer or coupled spreading frameworks, in which biological transmission and information exchange evolve through interconnected networks [32,140,141,142]. Information received by farmers may alter surveillance, preventive actions, and management decisions, which in turn feed back on disease transmission.

A particularly relevant example was provided by Yi et al., who constructed a multilayer network for foot-and-mouth disease transmission among beef cattle farms by combining animal movements, truck visits, and information-sharing interactions [32]. Their analysis showed that the structure and participation of the information-sharing layer can affect disease containment, illustrating how information exchange and biological transmission can interact within the same agricultural system. From a complementary perspective, Murray-Watson et al. explicitly coupled plant disease dynamics with grower decision-making and showed that disease-control outcomes depend strongly on the information and perceived risks used by growers when choosing control strategies [33]. These studies demonstrate that agricultural disease dynamics cannot always be separated from information-driven human responses.

Empirical studies further support the relevance of an information layer in agricultural disease management. Mueller et al. used social media to monitor corn and soybean diseases in the United States and showed that rapid information sharing helped track the northward spread of southern rust and provided early warning for field scouting [143]. Danielsen et al. analyzed information sharing around plant clinics in China and Zambia using social network analysis, demonstrating that the reach of plant-health information depends strongly on the structure of farmer communication networks [144]. In livestock systems, Rivera-Gomis et al. found that veterinarians are a major source of animal-health information for farmers and that combining multiple information channels can improve communication coverage [145].

These findings motivate coupled disease–information models for evaluating agricultural prevention and control strategies. Strong and timely information diffusion may facilitate earlier intervention and reduce opportunities for biological transmission, whereas weak or delayed coupling may allow outbreaks to progress before effective control measures are adopted. However, quantitative characterization remains challenging because biological transmission, information exchange, and behavioral responses are often recorded separately, at different spatial and temporal resolutions, making coupling strengths difficult to estimate from empirical data.

Beyond these direct spreading applications, complex agricultural systems also involve a wide range of biological, engineering, processing, monitoring, and control processes [146,147,148,149,150,151,152]. These include pathogen and disease mechanisms [153,154,155], postharvest preservation and microbial control [156,157,158,159,160,161,162,163,164,165,166,167], food processing and quality management [168,169,170,171,172], irrigation management [173,174], agricultural machinery and engineering technologies [175,176], and contaminant, pathogen, or residue detection [177,178,179,180,181,182]. Although these studies do not necessarily employ spreading models directly, they provide empirical measurements of system states, environmental conditions, interactions, and intervention processes that can support network reconstruction, model calibration, and spreading-control strategies.

## 6. Conclusions and Future Directions

Spreading dynamics on complex networks provide a general framework for describing the propagation of diseases, information, behaviors, and failures [1]. Extensions to multilayer [9], temporal [12], and higher-order networks [13] further broaden the ability of network models to represent the structural and dynamical complexity of agricultural systems [183,184,185,186,187]. As reviewed above, their successful application to agriculture depends not only on the choice of spreading model, but also on whether the underlying network representation, interaction mechanism, and available data adequately reflect the agricultural process of interest.

A first research direction is to develop more realistic representations of agricultural networks. Transportation, irrigation, trade, and seasonally varying activities can generate strongly heterogeneous and time-dependent interaction patterns [188,189,190,191,192]. Incorporating weighted, temporal, multilayer, and higher-order interactions may therefore improve the representation of transmission pathways and help identify vulnerable components or critical routes. Empirical characterization of transmission routes, such as the airborne dispersal of fungal spores in greenhouse crop systems, may provide direct information for reconstructing biologically meaningful spreading pathways [193]. Such extensions may also be useful for describing complex biological interactions in agricultural ecosystems, including microbial and microbiome dynamics [194,195,196,197,198,199].

A second direction is to better characterize the coupling between harmful spreading and beneficial information or control responses. Diseases and pests may propagate simultaneously with warnings, awareness, and behavioral responses, often through different contagion mechanisms [140,141,142]. Agricultural behaviors and technology adoption may also depend on spatial and farm-level heterogeneity, as illustrated by empirical studies of conservation-tillage adoption [200]. In practice, agricultural risk management involves pest forecasting, behavioral regulation, sensing, selective spraying, pesticide delivery, and other intervention technologies [201,202,203,204,205,206]. Multilayer and coupled-spreading models that jointly describe biological transmission, information diffusion, and control responses could therefore provide a useful framework for evaluating how the timing, strength, and targeting of interventions reshape spreading pathways and outbreak dynamics. More broadly, the diversity of biological, engineering, production, and monitoring processes in agriculture implies substantial heterogeneity in the mechanisms that spreading models need to capture [207,208,209,210].

A third direction is to strengthen the integration of spreading theory with agricultural sensing, engineering, and control technologies. Modern agricultural systems increasingly provide detailed observations of system states and potential intervention channels through sensing, imaging, monitoring, automation, and intelligent control. Representative examples include sensing, disease assessment, and contaminant detection [211,212,213,214,215,216,217,218,219,220,221,222,223,224,225,226,227,228,229,230,231], agricultural production and monitoring systems [232,233,234,235], irrigation and greenhouse management [236,237,238,239,240,241,242,243,244,245,246], agricultural machinery and mechanized production systems [247,248,249,250,251,252,253,254,255,256], and food processing, postharvest preservation, and safety monitoring [257,258,259,260,261,262,263,264,265]. Although these studies do not necessarily formulate spreading processes explicitly, they provide measurements of system states, environmental conditions, interactions, and control actions that can support network reconstruction, parameter estimation, model calibration, and intervention design.

Related agricultural engineering studies on physical, chemical, biological, processing, preservation, and microbial-control technologies provide additional experimentally characterized system responses and intervention mechanisms [266,267,268,269,270,271,272,273,274,275,276,277]. Incorporating such experimentally informed responses into network-based spreading models could help bridge the gap between theoretical risk prediction and practical intervention, allowing predicted spreading pathways to be linked more directly to available agricultural control measures.

A fourth direction is to combine mechanistic spreading models with data-driven and artificial-intelligence methods. Movement, trade, surveillance, environmental, and communication data can support the reconstruction and calibration of agricultural networks, while machine-learning methods can assist in inferring missing interactions, estimating transmission parameters, and predicting spreading risks [278]. Recent advances in smart agriculture and precision crop management further demonstrate the growing role of artificial intelligence in agricultural monitoring, disease diagnosis, decision support, and intelligent control [36,279]. Digital-twin and intelligent-equipment technologies also provide promising platforms for integrating real-time observations with model-based prediction and intervention [247,280]. Combining data-driven approaches with mechanistic spreading models may improve predictive performance while retaining physical interpretability, although uncertainties arising from incomplete observations and heterogeneous data sources should be explicitly considered [281]. In particular, deep-learning methods for pest and disease detection and control may provide useful inputs for identifying outbreak states, updating spreading models, and supporting adaptive intervention strategies [282].

Overall, spreading theories developed for social and contact networks provide a useful conceptual and methodological foundation for studying propagation processes in complex agricultural systems. Future progress will require closer integration among realistic network representations, process-specific dynamical models, empirical agricultural data, and practical intervention technologies. Such integration may enable spreading models to move beyond qualitative description toward quantitative risk assessment, prediction, and targeted control in real-world agricultural systems.

## Figures and Tables

**Table 1:** Applicability and limitations of representative mathematical approaches for spreading dynamics.

Approach	Main Assumption	Structural Resolution	Agricultural Applicability	Main Limitation
MF	Homogeneous mixing	Population level	Large-scale or aggregate spreading	Neglects farm-level, spatial, and structural heterogeneity
HMF	Nodes grouped by degree	Degree classes	Spreading with heterogeneous connectivity among farms or regions	Ignores node-specific structure and local correlations
QMF	Static known network	Node level	Spreading on empirical farm, contact, or movement networks	Sensitive to network completeness; limited for temporal interactions
DMP	Locally tree-like propagation	Edge level	Transmission along explicit contact or movement pathways	Less accurate for strong clustering, loops, or repeated contacts
PA	Local pair correlations	Pair level	Local transmission with correlated states between connected units	Computational complexity increases with structural heterogeneity
BP	Independent transmission on locally tree-like networks	Degree/edge level	Final outbreak size and invasion probability for irreversible spreading	Less suitable for strong clustering, temporal contacts, or correlated transmission
EBCT	Edge-based transmission	Degree/edge level	SIR-like and reinforcement-based spreading on heterogeneous networks	Requires explicit degree structure and transmission or adoption rules

**Table 2:** Representative micro-level mechanisms underlying spreading processes in agricultural networks.

Scenario	Nodes and Edges	Propagated State	Local Mechanism	Emergent Dynamics
Crop disease	Plants, fields, or farms linked by proximity or transport	Pathogen, infection	Pathogen transfer through dispersal or movement	Spatial outbreaks and epidemic spread
Livestock disease	Animals or farms linked by contact or movement	Infection	Transmission through contact or animal movement	Farm-to-farm epidemics
Technology adoption	Farmers linked by social ties	Knowledge, adoption	Peer influence and social reinforcement	Adoption cascades
Agricultural information	Farmers or organizations linked by communication	Warnings, knowledge	Interpersonal or digital information exchange	Network-wide information diffusion
Disease–information coupling	Farms or farmers connected by disease and information layers	Infection, awareness	Information modifies preventive or control behavior	Coupled disease-behavior dynamics

**Table 3:** Applicability and practical limitations of spreading models across representative agricultural scenarios.

Scenario	Process	Suitable Model	Practical Limitations
Crop disease	Pathogen transmission	SIR/SIS and spatial models	Uncertain transmission rates; environmental heterogeneity
Pest invasion	Spatial dispersal	Spatial and agent-based models	Uncertain dispersal pathways; incomplete observations
Livestock disease	Farm-to-farm transmission	SIR and temporal models	Incomplete movement data; temporal heterogeneity
Technology adoption	Complex contagion	Threshold and social network models	Behavioral heterogeneity; uncertain adoption thresholds
Agricultural information	Information spreading	Social network diffusion models	Incomplete communication data; unobserved interactions
Disease–information coupling	Coupled spreading	Multilayer models	Uncertain coupling strength; heterogeneous data sources

## Data Availability

No new data were created or analyzed in this study. Data sharing is not applicable to this article.
